# Successful reduction of urinary catheter placement for correct and incorrect indications after introduction of a prevention bundle

**DOI:** 10.1017/ash.2024.27

**Published:** 2024-05-02

**Authors:** Michela Cipriani, Matthias Schlegel, Marianne Schwark-Bähler, Samuel Henz, Philipp Kohler, Werner C. Albrich

**Affiliations:** 1 Departement of Infectious Diseases, Infection Prevention and Travel Medicine, Cantonal Hospital St. Gallen, St. Gallen, Switzerland; 2 Department of General Internal Medicine, Cantonal Hospital St. Gallen, St. Gallen, Switzerland

## Abstract

The prospective before-after quality improvement study was to assess bundle effectiveness to reduce urinary catheter days and prevent associated complications. All patients with preexisting or new urinary catheters in a regional hospital in Switzerland were included. We showed a reduction of catheter days, incorrect urinary catheter indications, and most strikingly formally correct indications.

## Introduction

The presence of urinary catheters and catheter dwell times are the main risk factors for potential infectious and noninfectious complications of urinary catheters and are associated with increased morbidity, mortality, and healthcare costs.^1^ Catheter-associated urinary tract infections (CAUTIs) rates range from 0.2 to 4.8 per 1,000 catheter days.^1^ Not only are CAUTIs frequent but also one of the best preventable nosocomial infections. According to a recent meta-analysis, about 54% of CAUTIs are considered preventable.^2^

In our 2015 retrospective analysis of all hospitalized patients at the regional hospital of Rorschach in Switzerland,^3^ catheter exposure was 13.2 catheter days/10,000 hospitalization days, which was shorter than baseline values in a Swiss multicenter intervention study.^1^

Several studies have shown that the implementation of intervention bundles focusing on the reduction of unnecessary catheter use, proper insertion techniques, and safe catheter maintenance can help in reducing CAUTIs.^4–7^ However, little is known whether bundles can reduce urinary catheter utilization beyond the reduction of incorrect indications. To assess the effectiveness of our bundle to reduce urinary catheter days and to prevent catheter-related infectious and noninfectious complications, we conducted a before-after study at the same hospital (as in 2015^3^) evaluating both correct and incorrect indications for urine catheterization.

## Methods

This prospective before-after quality improvement study was carried out at the 62-bed regional hospital of Rorschach in Switzerland between October 2018 and May 2019. The prevention bundle consisted of 3 evidence-based measures: training of hospital personnel emphasizing adequate indications for catheterization, catheter insertion only according to defined criteria with daily reevaluation, and training of personnel for safe insertion and handling of bladder catheters. The primary objective of the study was to assess the effectiveness of the bundle regarding catheter insertion frequency and days of catheter dwell time. The secondary objectives were to assess the bundle effectiveness on CAUTI and noninfectious catheter complications.

We prospectively included all consecutive patients with preexisting or newly placed urinary catheters, which remained in place for at least 24 hours. We defined catheterization as indicated if any of the 6 major indications of the “progress! Urinary catheter safety” study, a pilot program in Switzerland from 2015 to 2018 with the aim to reduce CAUTI rates and noninfectious complications, were fulfilled (Supplemental Table 1 (online)).^8^ All other catheterizations were considered not indicated. We defined CAUTI according to established criteria by the US Centers for Disease Control and Prevention.^9^

To account for potential differences in disease severity, data on the diagnosis-related group-case mix index (CMI) were calculated from the total cost weight divided by the total of treatment cases incurred during a period of time. Nursing expenses were obtained from the hospital’s financial department. Catheter cases per number of discharged patients, catheter days per bed days, catheter dwell time, indications for catheterization, as well as CAUTIs and noninfectious complications were compared between the 2-month period before (October 15, 2018–December 14, 2018) and the 4-month period after (January 21, 2019–May 10, 2019) a hospital-wide 5-week implementation phase of the catheter bundle. Categorical variables were compared with χ^2^ tests or Fisher’s exact test, as appropriate, using OpenEpi (Version 3.01, Atlanta, USA, 2013). The effective reduction of catheter insertion rate was calculated by subtracting the observed postintervention insertions from the expected insertions using the following formula:

[(insertions_pre_/discharges_pre_) * discharges_post_ – insertions_post_]/discharges_post_

*P* values ≤ .05 were considered significant.

## Results

A total of 193 catheter cases were identified: 101 among 510 discharged patients (19.8%) before and 92 among 894 discharges (10.3%) after the intervention (*P* < .001). Baseline characteristics for patients with captured catheter cases were similar in both periods except for fewer admissions to the nephrology department (Table 1). The CMI (0.842 vs 0.863) and the nursing expenses (CHF 4'903 vs CHF 4'912) were similar for the 510 and 894 patients hospitalized during the before and after periods, respectively, indicating similar patient and treatment characteristics during both periods.

**Table 1.** Patient baseline characteristics

	Before (*n* = 101)	After (*n* = 92)	*P* value
**Age** (mean, [range]) in years	78 [28–97]	80 [34–97]	.6
**Gender (** ***n*** **)**			
Male	51 (50%)	44 (48%)	.8
**Departments (** ***n*** **)**			
Internal medicine	58 (58%)	56 (61%)	.8
Orthopedics	20 (20%)	19 (21%)	.9
Surgery	13 (13%)	15 (16%)	.6
Nephrology	10 (10%)	2 (2%)	**.03**
**Length of stay**			
(median, [IQR]) in days	11.6 [2–47]	12.7 [3–47]	.8
**Catheter insertion**			
Time since catheter insertion if known (median) days	3.2	2.3	.9
**Case mix index**	0.842	0.863	.9
**Nursing expenses (CHF)**	4,903	4,912	.5

Overall, there was a significant reduction of catheter days during the postintervention period (1,898/10,000 hospital bed days vs 1,269/10,000 hospital bed days; *P* < .001). There was a nonsignificant trend for an increase of catheter dwell time from 5.8 days to 7.4 days (*P* = .16).

**Figure 1.**
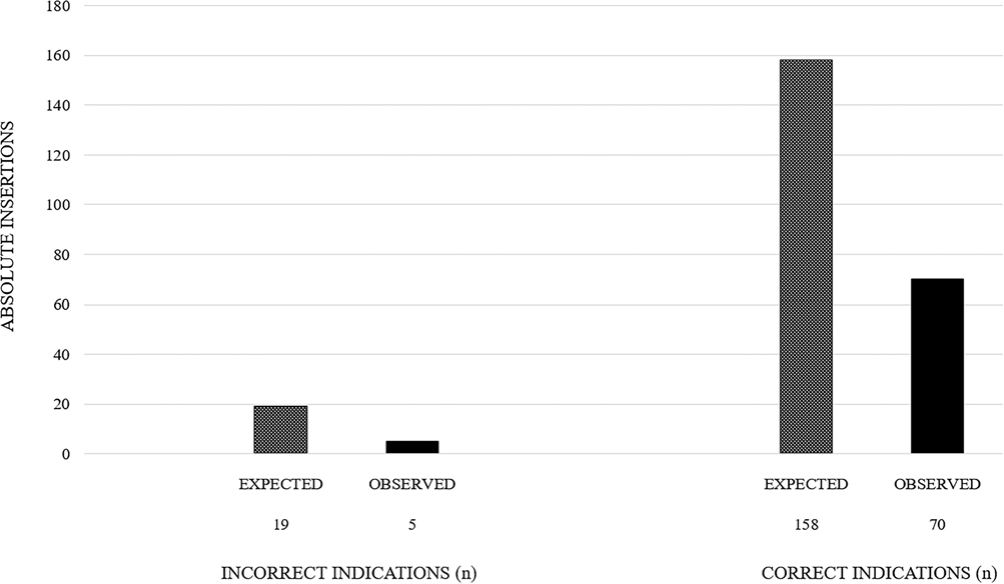
Effective reduction of catheter insertions for incorrect and correct indications by applying the intervention bundle.

From pre- to postintervention, the proportion of incorrect catheter insertions decreased significantly (11/510 [2.2%] vs 5/894 [0.6%], *P* = .01) as did the proportion of correct catheter insertions (90/510 [17.6%] vs 87/894 [9.7%], *P* < .001). Therefore, the effective reduction of catheter insertions for correct indications was larger (71/894 discharges) than for incorrect indications (14/894 discharges). Among patients with new catheters, there was a nonsignificant reduction of incorrect indications (11/101 vs 5/92, *P* = .17, Figure 1).

Incorrect indications during preintervention and postintervention phases were catheterization for incontinence (5% vs 0%, *P* = .03) and for urinary tract infection (3% vs 3%, *P* = .9). The most frequent correct indications for catheterization during preintervention and postintervention phases were urinary retention (41% vs 46%, *P* = .6), followed by urine monitoring (35% vs 39%, *P* = .7), and surgery (15% vs 18%, *P* = .6).

The overall risk of CAUTIs was low both before (3/101, 3%) and after intervention (2/92, 2%) (*P* = .9). Most CAUTIs (4/5, 80%) were related to preexisting urinary catheters, which were not removed on admission. A noninfectious complication (bleeding) was observed in only one patient in the postintervention period.

## Discussion

During the postintervention period, we showed a significant reduction of catheter days and a reduction of incorrect indications, which was achieved also in previous intervention studies.^10^ These findings can be attributed to the increased knowledge and awareness toward catheter use. Surprisingly, during the postintervention period, we also found a significant reduction of correct indications for catheter insertion, which, to our knowledge, has not been described yet. Importantly, the prevented number of catheter insertions was larger through the reduction of correct catheter indications than through the reduction of incorrect indications.

This finding demonstrates that also a formally adequate indication for catheter insertion has a large potential to be avoided with subsequent reduction of catheter-associated complications. Consequently, future intervention studies should aim to reduce the overall use of urinary catheters not limited to inappropriate indications, as present indications for urinary catheter insertion may be too broad and should be reassessed. In this study, there was no relevant difference concerning infectious and noninfectious catheter-associated complications after the intervention, which is consistent with previous findings^1^ and most likely due to the preexisting low rate in the before period.

Our study has some limitations. This was a single-center study with a relatively small number of events. Since it was a hospitalwide study, there were no control wards without intervention. On the other hand, stable CMI and nursing expenses between both periods argue against differences in patient population or other imbalances.

In conclusion, this intervention bundle led to a significant reduction of catheter cases and catheter days, due to reductions of insertions both with incorrect indications and most strikingly—and unexpectedly—with formally correct indications. This suggests, first, a huge potential of avoiding formally indicated but likely not necessary catheterizations, underlining the importance of looking for alternatives to catheterization, and second, that not all correct indications should automatically result in catheter insertion.

## Supporting information

Cipriani et al. supplementary material Cipriani et al. supplementary material

## Supplementary material

For supplementary material accompanying this paper visit http://doi.org/10.1017/ash.2024.27. click here to view supplementary material
