# Supplementary material for: Successful reduction of urinary catheter placement for correct and incorrect indications after introduction of a prevention bundle
Source: Antimicrob Steward Healthc Epidemiol. 2024 May 2;4(1):e71. doi: 10.1017/ash.2024.27 (PMC11077593; doi:10.1017/ash.2024.27)
Supplement: Cipriani et al. supplementary material [file S2732494X24000275sup001.doc]

Suppl. Table 1

**List of correct indications for urinary catheter insertion per the Swiss ‘Progress! Safe urinary catheterization’ programme [8]**

| **INDICATION** | **SPECIFICATION** |
| --- | --- |
| URINARY RETENTION | - Acute urinary retention regardless of aetiology - Symptomatic chronic outlet obstruction plus >300 mL residual urine |
| MEASUREMENT OF URINE VOLUME/FLUID BALANCE | - At regular intervals (hourly or as defined by hospitals) plus direct consequence on treatment of patients - Fluid balance if patient weight not measurable on a daily basis |
| SURGERY | - Prolonged surgery (>4 h) - Peri-interventional: need for empty bladder during surgery, removal of catheter after surgery necessary if no other indication present - Surgery in urogenital or pelvic floor region - Epidural/peridural anaesthesia |
| PRESSURE ULCERS PLUS  URINARY INCONTINENCE | - Stage III or IV pressure ulcers or skin transplants in sacral/perineal region plus urinary incontinence after exhaustion of alternative strategies for urinary management |
| PROLONGED IMMOBILIZATION | - Immobilization for medical reasons, especially for pain management, after exhaustion of alternative strategies for urinary management |
| PALLIATIVE CARE PLUS COMFORT | - Palliative care plus abnormal bladder function plus/or inability for regular voiding after exhaustion of alternative strategies for urinary management - High burden of suffering plus wish of informed patient (or relatives) |
